# Plant Functional Diversity Can Be Independent of Species Diversity: Observations Based on the Impact of 4-Yrs of Nitrogen and Phosphorus Additions in an Alpine Meadow

**DOI:** 10.1371/journal.pone.0136040

**Published:** 2015-08-21

**Authors:** Wei Li, Ji-Min Cheng, Kai-Liang Yu, Howard E. Epstein, Liang Guo, Guang-Hua Jing, Jie Zhao, Guo-Zhen Du

**Affiliations:** 1 State Key Laboratory of Soil Erosion and Dryland Farming on the Loess Plateau, Northwest A&F University, Yangling, 712100, China; 2 Institute of Soil and Water Conservation of Chinese Academy of Sciences & Ministry of Water Resource, Yangling, 712100, China; 3 Department of Environmental Sciences, University of Virginia, Charlottesville, Virginia, 22904–4123, United States of America; 4 College of Animal Science and Technology, Northwest A&F University, Yangling, 712100, China; 5 School of Life Sciences, Lanzhou University, Lanzhou, 730000, China; National Taiwan University, TAIWAN

## Abstract

Past studies have widely documented the decrease in species diversity in response to addition of nutrients, however functional diversity is often independent from species diversity. In this study, we conducted a field experiment to examine the effect of nitrogen and phosphorus fertilization ((NH_4_)_2_ HPO_4_) at 0, 15, 30 and 60 g m^-2^ yr^-1^ (F0, F15, F30 and F60) after 4 years of continuous fertilization on functional diversity and species diversity, and its relationship with productivity in an alpine meadow community on the Tibetan Plateau. To this purpose, three community-weighted mean trait values (specific leaf area, SLA; mature plant height, MPH; and seed size, SS) for 30 common species in each fertilization level were determined; three components of functional diversity (functional richness, FRic; functional evenness, FEve; and Rao’s index of quadratic entropy, FRao) were quantified. Our results showed that: (i) species diversity sharply decreased, but functional diversity remained stable with fertilization; (ii) community-weighted mean traits (SLA and MPH) had a significant increase along the fertilization level; (iii) aboveground biomass was not correlated with functional diversity, but it was significantly correlated with species diversity and MPH. Our results suggest that decreases in species diversity due to fertilization do not result in corresponding changes in functional diversity. Functional identity of species may be more important than functional diversity in influencing aboveground productivity in this alpine meadow community, and our results also support the mass ratio hypothesis; that is, the traits of the dominant species influenced the community biomass production.

## Introduction

In terrestrial plant communities, fertilization via nutrient deposition and agricultural inputs often leads to decreases in plant diversity and shifts in community composition [1, 2]. Numerous experiments of nutrients addition, particularly in grasslands, also reported nearly ubiquitous negative productivity–diversity relationships [1–4]. Although this pattern is well documented [5], attempts to link biodiversity to aboveground productivity following fertilization are rare (but see [6, 7]). The causes of fertilization-induced species loss for community productivity, therefore, remain unclear. Comprehensive understanding the changes of plant diversity is especially critical to developing policies that minimize the loss of species under future global climate change scenarios.

Plant diversity is a multifaceted concept that can be measured with a variety of indicators [8]. Traditionally, most studies on plant diversity have mainly focused on species diversity (measured as species richness and/or species evenness) [9], and other components of diversity have frequently been underestimated. In particular, what remains less investigated is functional diversity (i.e., range, values and distribution of key morphological and physiological traits in a given community or as those components of plant diversity that influence ecosystem functioning) [10, 11]. In fact, the mechanisms by which communities respond to environmental change (e.g., fertilization) are expected to depend on the functional traits of the species [10]. A growing body of evidence has shown that functional diversity is directly linked with ecosystem processes (e.g., productivity) [11–13]. Although functional diversity has received ample theoretical attention [14, 15], still there is little empirical data showing that how functional diversity varies along an artificial fertilization level. In addition, the relationship between species diversity and functional diversity is central in identifying the diversity effects on ecosystem functioning [16]. The negative effect of fertilization on species diversity is well documented [1, 2], but the changes in functional diversity may not simply follow the decrease in species diversity [17, 18]. De Bello et al. [19] also demonstrated that the increased species diversity with increased environmental pressure was not followed by similar trajectories for functional diversity, and the species diversity and functional diversity were independent of each other. Therefore, examining functional diversity has been proposed as a promising method to identify the effects of species loss on ecosystem functioning following fertilization [16, 18]. In addition, we use aboveground productivity as a proxy of ecosystem functioning, and the other functions of ecosystems such as biogeochemical cycles, invasion resistance, stability in the face of disturbance are not being taken into consideration in this study.

From both experimental and observational studies, two mutually nonexclusive hypotheses have been proposed to explain the effect of species diversity on ecosystem processes. The “mass ratio hypothesis” states that the extent to which the traits of a species affect ecosystem functioning is likely to be related to the contributions of the species to the biomass of the community [20]. This hypothesis was confirmed by some studies that account for ecosystem functions such as primary productivity [21] and nitrification [22]. Conversely, the “diversity hypothesis” postulates that the diversity of functional traits (the degree to which trait values differ between species) within a community can affect ecosystem processes [23]. The high functional diversity may allow for a more complete use of resources among species, thereby improving biomass production [11, 14]. Plants communities with high species richness may also allow for a high stability in response to disturbances because of function redundancy [24–26]. Although the “diversity hypothesis” has been supported by some studies [23], test of functional diversity indices are still rare [5, 27]. Recent studies have shown that the “mass ratio hypothesis” and the “diversity hypothesis” are not mutually exclusive, and community-weighted means of trait values and functional trait diversity may together affect ecosystem processes in semi-natural grasslands [27, 28].

The Tibetan Plateau is the youngest and highest plateau in the world. Alpine meadows comprise the representative vegetation on the plateau, and they are also very fragile and sensitive ecosystems due to changes in global climate and land use [29]. In the past few decades, the alpine meadow communities have experienced an unprecedented input of external nutrients, due to an increase of human pressure and livestock [30]. Such disturbance (e.g. gaps made by feet of domestic stock, small heaps made by pikas), accompanied by overgrazing has important effects on community composition and structure [31]. Previously, a series of fertilization experiments were conducted in an alpine meadow on the Tibetan Plateau, China to better understand the potential mechanisms of species loss due to fertilization [32–34]. However, to our knowledge, no studies so far have explored the effects of fertilization on functional diversity in this region.

In this study, we tested the following predictions: (i) following fertilization functional diversity may increase (due to stronger competition and niche differentiation) [6, 35], decrease (due to environmental filtering or species loss) [18, 36] or remain stable due to a balance between the two antagonistic mechanisms; (ii) functional diversity may have a stronger positive correlation with community productivity than species diversity because it is directly linked to community biomass production[11–13]; (iii) the community-weighted means of trait values (CWM) simplifies the community into an average trait value that is strongly determined by the functional trait values of the dominant species; changes in CWM in response to fertilization may predominantly drive community biomass production [20–22].

## Material and Methods

### Study site

The experiment was conducted at the Research Station of Alpine Meadow and Wetland Ecosystems of Lanzhou University (N 33°58′, E101°53′) on the eastern Tibetan plateau, 3500 m a.s.l., Gansu, China. The yearly average temperature is 1.2° C, ranging from -10° C in January to 11.7° C in July, with ~ 270 frost days. Annual precipitation, averaged over the last 35 years, is 620 mm (most in summer). The annual cloud-free solar radiation is ~ 2580 h [34]. The vegetation, typical of Tibetan alpine meadows, is dominated by clonal *Kobresia* spp., *Elymus nutans*, *Festuca ovina*, *Poa poophagorum*, *Agrostis* spp., *Saussurea* spp. and *Anemone rivularis*. The experimental site has been overgrazed in the past, but has been fenced by wire netting during the growing season since May 2007, with grazing limited to the non-productive winter months (October to April in the following year) [34].The average soil organic C(%), available N(kg^−1^), available P(kg^−1^) and PH is 1.6, 16.2, 2.1 and 7.1, respectively before applying fertilizers.

### Experimental design

Thirty-six 4 × 10-m^2^ plots composed of four fertilization levels with nine replicates were distributed in nine columns and four rows with a randomized block design. Each plot was separated from the others by a 2-m buffer strip. The fertilization treatment was generated with different amounts of (NH_4_)_2_ HPO_4_ fertilizer applied annually from 2007 to 2010. Fertilizer applications of 0, 15, 30 and 60 g m ^-2^ yr ^-1^ are hereafter referred to as F0, F15, F30 and F60, which corresponds to 0, 3.15, 6.3, and 12.6 g N m^-2^ yr ^-1^ and 0, 3.5, 7.0 and 14.0 g P m^-2^ yr^-1^, respectively. The fertilizer was applied at the beginning of the growing season (usually in the middle of May) each year during drizzly days to avoid the need for watering [34]. Each plot was separated into two subplots: a 4 × 4 m subplot for community investigation and biomass harvest, and a 4 × 6 m subplot for individual plant sampling.

#### Community measurements

Community measurements were conducted from 2 to 4 Sept 2010. One 0.25 m^2^ quadrat was harvested from the 4 × 4 m subplot in each plot. The quadrat location was randomly selected with the constraint that it was at least 0.5 m from the margin to avoid edge effects. The number of ramets per species was recorded, and then the ramets were clipped (about 2 cm residue was left to avoid death) and brought to the laboratory. For clonal species, an individual plant was defined as a group of tillers connected by a crown [34]. All samples were dried at 80°C for 48 h, and weighed to the nearest 0.01 g. Community aboveground biomass was calculated by summing all dried biomass of harvested individuals within a quadrat.

#### Plant functional trait measurements

Following Cornelissen et al. [37], we measured functional traits on the 30 most common species in each fertilization level in early September 2010. These species represented 85–95% of the peak standing biomass and 80–90% of the vegetation cover of the total plant community in the studied plots. The Leaf-Height-Seed scheme proposed that three key functional traits, specific leaf area (SLA), mature plant height (MPH) and seed size (SS), could capture the main axes of variation in ecological strategies among species [38]. Therefore, we chose SLA, MPH and SS to estimate key dimensions of ecological strategies.

We randomly sampled 2 individuals and 6 mature leaves (3 leaves per individual) at flowering time for each of the 30 species in each 4 × 6 m subplot to measure SLA and MPH, respectively [37]. That is, 18 individuals and 54 mature leaves were measured for each of the 30 species in each fertilization level. Mature plant height is the perpendicular distance between the upper foliage boundary and ground Leaves were scanned to measure leaf area in the field, and were placed in paper bags and dried in the sun. Leaf samples were oven-dried at 80°C for 48 h in the laboratory and their dry masses were measured on a balance with an accuracy of 10^−4^ g (Acculab Lt-320; Acculab, Measurement Standards Inc., Danvers, MA, USA). We also collected approximately 800 mature seeds from 20–30 individuals for each species in each fertilization level from Jul to Oct in 2010. Seeds were air-dried and kept in the laboratory (15°C). Seed mass was defined as mass of the embryo and endosperm, including seed coat. Structures having the function of contributing to dispersal (appendages, fruit coat in some cases) were not included in the seed mass. Three replicates of 100 seeds were weighed on a balance with an accuracy of 10^−4^ g for each species to obtain the seed mass per 100 seeds.

### Data analysis

From the vegetation harvest data, three indices were selected to estimate species diversity according to Casanoves et al. [39] (Table 1). We calculated the means of three functional traits for each species in each fertilization level. The means of measured traits in each fertilization level are listed in S1 Table. Moreover, our study mainly focuses on comparing values of functional diversity and the CWM trait between different fertilization levels based on inter-specific trait variability, rather than intra-specific trait variability.

**Table 1:** Species diversity and functional diversity measures.

Index	Formula	Terms meaning	Reference
**Species richness**	*S* = N	N: number of species	Casanoves et al. (2011)
**Shannon–Weiner diversity index**	$H'=-\sum_{i=1}^{S}P_{i}\;\log_{2}P_{i}$	P_i_: relative abundance of species i	Casanoves et al. (2011)
**Evenness index**	$E=\frac{H\prime }{\ln(S)}$	*H*′: Shannon–Weiner diversity index	Casanoves et al. (2011)
**Functional richness (FRic)**	Quickhull algorithm		Villéger et al.(2008)
**Functional evenness (FEve)**	$FEve=\frac{\sum_{i=1}^{S-1}\min({PEW}_{i},\frac{1}{S-1})-\frac{1}{S-1}}{1-\frac{1}{S-1}}$	Partial weighted evenness: ${PEW}_{i}=\frac{{EW}_{bl}}{\sum_{b=1}^{S-1}{EW}_{bl}}$ Weighted evenness: ${EW}_{bl}=\frac{d_{ij}}{P_{i}+P_{j}}$ bl: branch length	Villéger et al.(2008)
**Rao index (FRao)**	$FRao=\sum_{i-1}^{S-1}\sum_{j=i+1}^{S-1}d_{ij}p_{i}p_{j}$	*d*_*ij*_: Euclidian dissimilarity between the traits of each pair of species i and j: $d_{ij}=\sum_{t=1}^{T}{\left(x_{tj}-x_{ti}\right)}^{2}$ x_ti_: tth trait value of i_th_ species-T: number of traits	Mouchet et al.(2010)
**community-weighted mean trait values (CWM)**	$CWM=\sum_{i=1}^{S}P_{i}\times {trait}_{i}$	trait_i_: is the trait value of species i	Garnier et al.(2004)

Following Garnier et al. [21], the community-weighted mean trait values for each trait were calculated for every sample using species mean trait values and species relative cover (Table 1). Although various indices have been proposed to measure the functional diversity of a community, there is still no consensus on which are most suitable. Villéger et al. [17] recently suggested that some indices of functional diversity are redundant and they recommended using three independent components of functional diversity—functional richness (FRic), functional evenness (FEve) and functional divergence (FDiv) (Table 1). Additionally, Rao’s index of quadratic entropy (FRao) has been widely used to indicate functional divergence of traits as it includes variance and functional dispersion and is strongly correlated with FDiv [40]. In this study, we chose FRic, FEve and FDrao to examine how different components of functional diversity responded to fertilization. We used the FDiversity software program to calculate FRic, FEve and FDiv using a Euclidean distance and an average linkage [39] after the traits were standardized to ensure equal contribution of each trait.

We assessed the relationship between species diversity and functional diversity by using correlation analysis. We used one-way ANOVA to test the effect of fertilization on plant species diversity (species richness, Shannon-Wiener diversity index, evenness index), the CWM traits and functional diversity (FRic, FEve and FRao). Then, we performed linear regression modeling to test the relationships between community biomass and species diversity, the CWM traits and functional diversity. These statistical analyses were performed using the R 3.2.0 software (R Development Core Team, 2011).

## Results

### Relationship between species diversity and functional diversity

Pearson correlation analysis showed that the indices of species diversity (richness, Shannon-Wiener diversity index and evenness) were robustly correlated among them (Table 2). Three functional diversity indices (FRic, FRao and FEve) were independent of each other (Table 2). In addition, species diversity and functional diversity were also independent of each other (Table 2).

**Table 2:** Pearson correlations among the considered diversity measurements. Fric, functional richness; FRao, Rao index.

	Fric	FRao	FEve	Species richness	Shannon–Weiner diversity index
**Frao**	-0.017				
**FEve**	0.005	-0.169			
**Species richness**	0.121	0.245	-0.231		
**Shannon–Weiner diversity index**	0.232	0.123	-0.153	**0.830**	
**Species evenness**	0.018	0.142	-0.217	**0.722**	**0.648**

FEve, functional evenness. Values in bold indicate a significant (p <0.05) correlation.

### Effects of fertilization on aboveground biomass, species diversity, functional diversity and CWM traits

**Fig 1:**
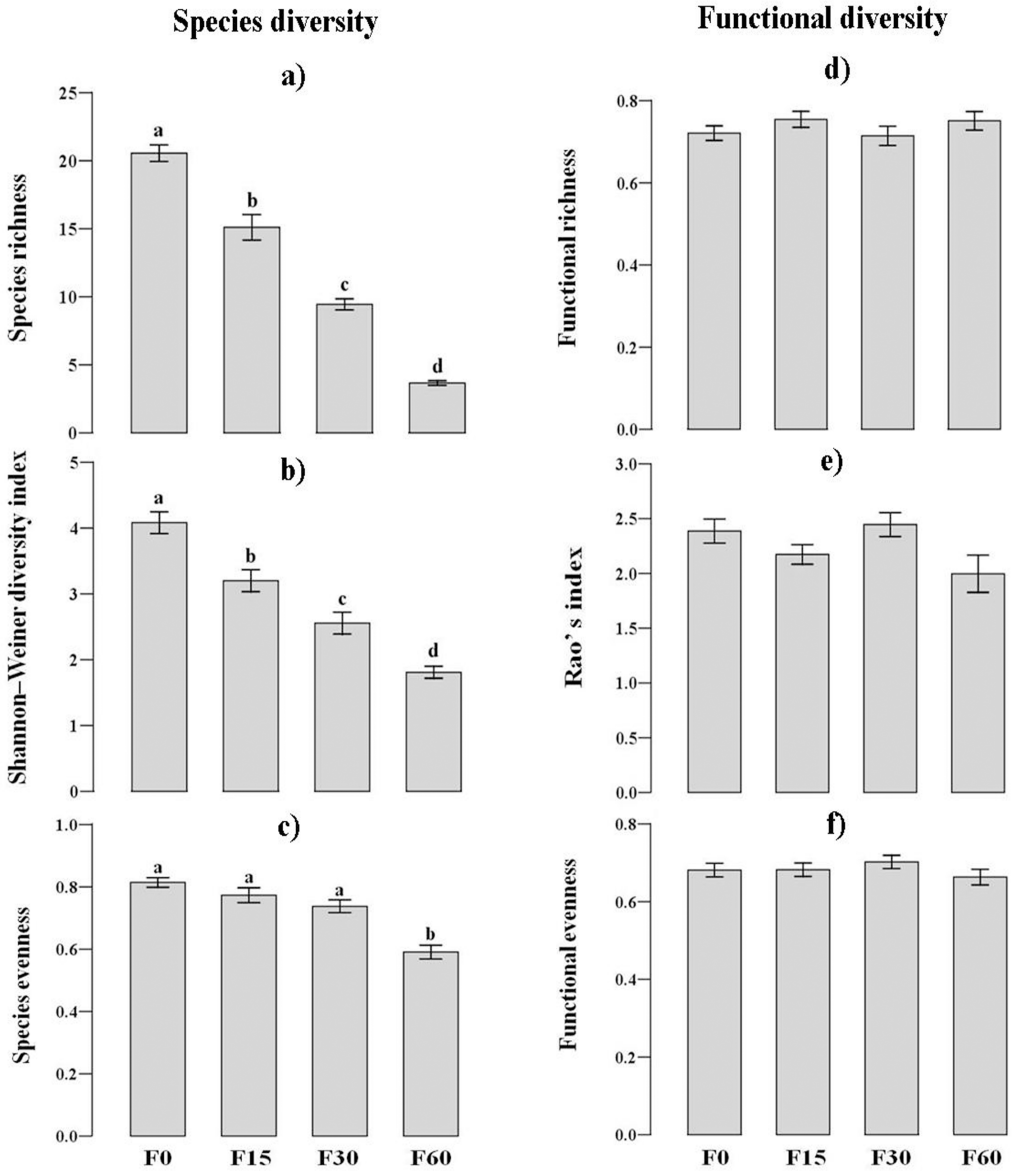
The changes of species diversity (a, b and c; n = 9) and functional diversity (d, e and f; n = 9) along the fertilization gradients. F0, F15, F30, and F60 represent (NH_4_)_2_HPO_4_ fertilizer applications of 0, 15, 30 and 60 g m^-2^ yr^-1^. Significant differences indicated by dissimilar letters above each bar were determined using Tukey’s honestly significant difference (HSD) test (P< 0.05) after one-way ANOVA.

**Fig 2:**
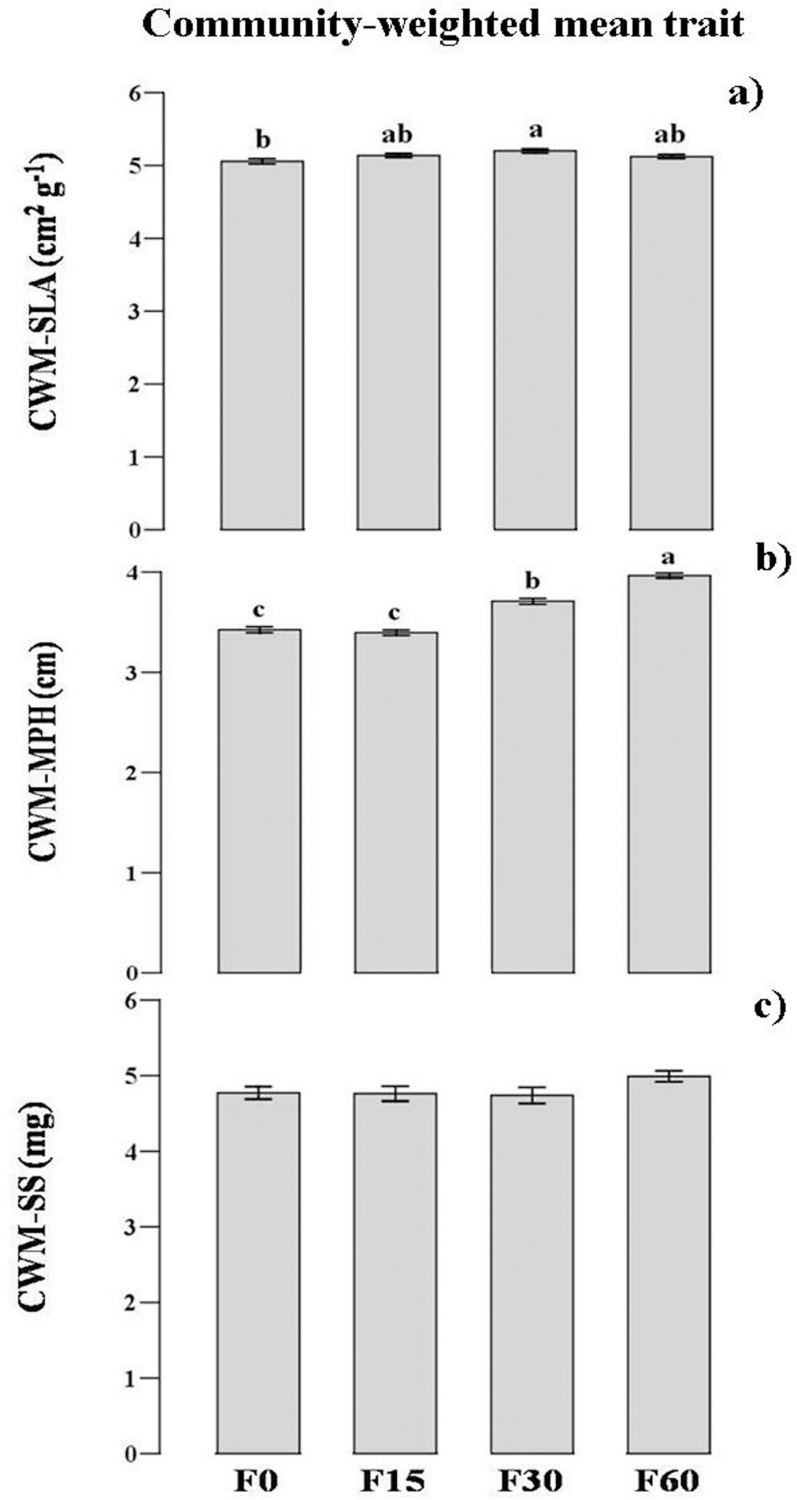
The changes of CWM trait (n = 9) along the fertilization gradients. CWM, community-weighted mean trait values; SLA, leaf area per unit dry mass; MPH, mature plant height; SS, seed size. F0, F15, F30, and F60 represent (NH_4_)_2_HPO_4_ fertilizer applications of 0, 15, 30 and 60 g m^-2^ yr^-1^. Significant differences indicated by dissimilar letters above each bar were determined using Tukey’s honestly significant difference (HSD) test (P< 0.05) after one-way ANOVA.

Aboveground biomass increased by 19–41% from low- to high-fertilized plots relative to control (F = 41.1, p < 0.001). Species richness decreased by 25–85% in fertilized plots relative to control (Fig 1A). Shannon-Wiener diversity index decreased by 20–55% in fertilized plots relative to control (Fig 1B). The species evenness decreased by 5–31% in fertilized plots relative to control (Fig 1C). FRic, FRao and FEve were not affected by fertilization (Fig 1D, 1E and 1F).The CWM-SLA and CWM-MPH show an increase with fertilization (Fig 2A and 2B), and the CWM-SS did not show any significant change along the fertilization level (Fig 2C).

### Relationship between biodiversity and community biomass

**Fig 3:**
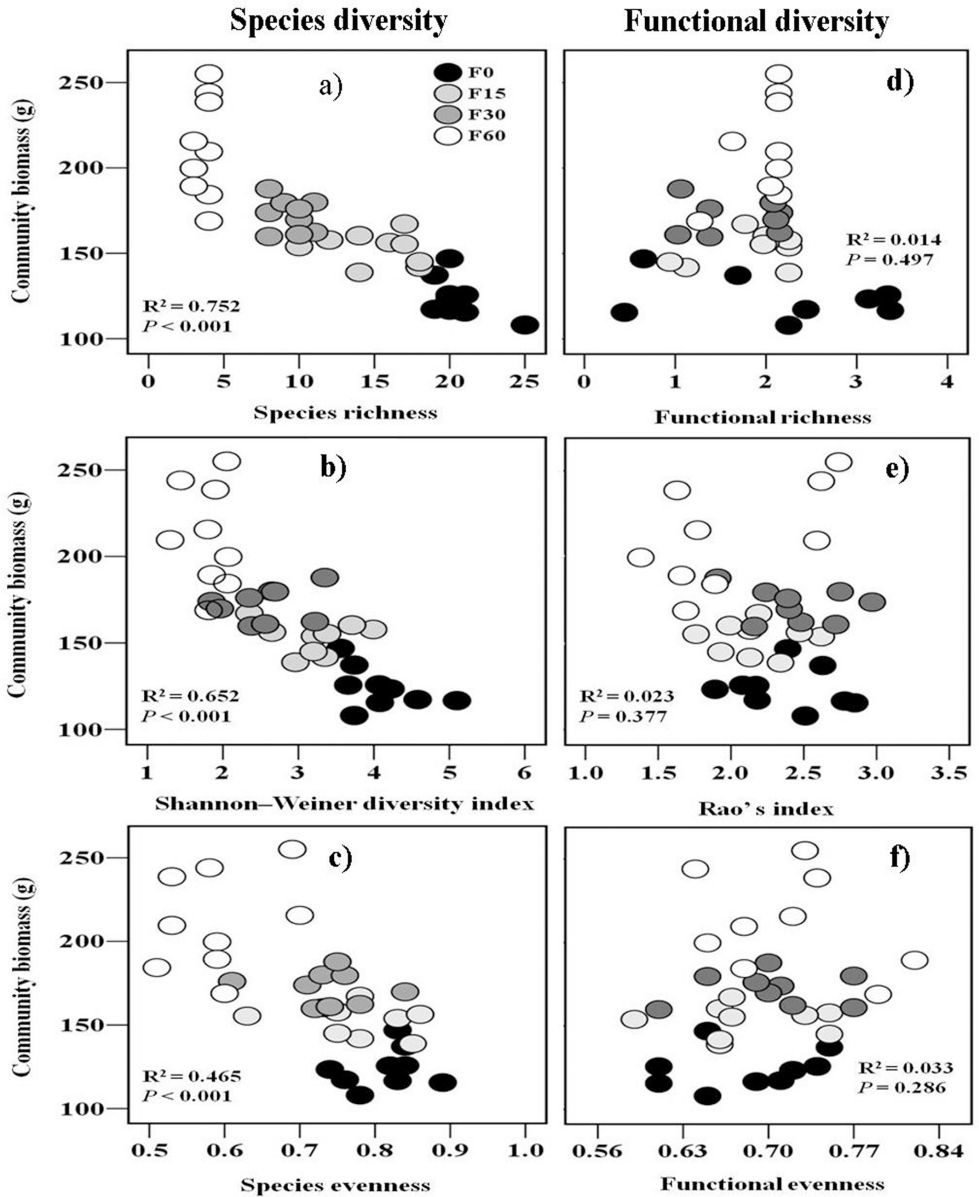
The relationships between community biomass (n = 9) and species diversity (a,b and c; n = 9) or functional diversity(d, e and f; n = 9). R^2^and P value was estimated from a linear regression model. F0, F15, F30, and F60 represent (NH_4_)_2_HPO_4_ fertilizer applications of 0, 15, 30 and 60 g m^-2^ yr^-1^.

**Fig 4:**
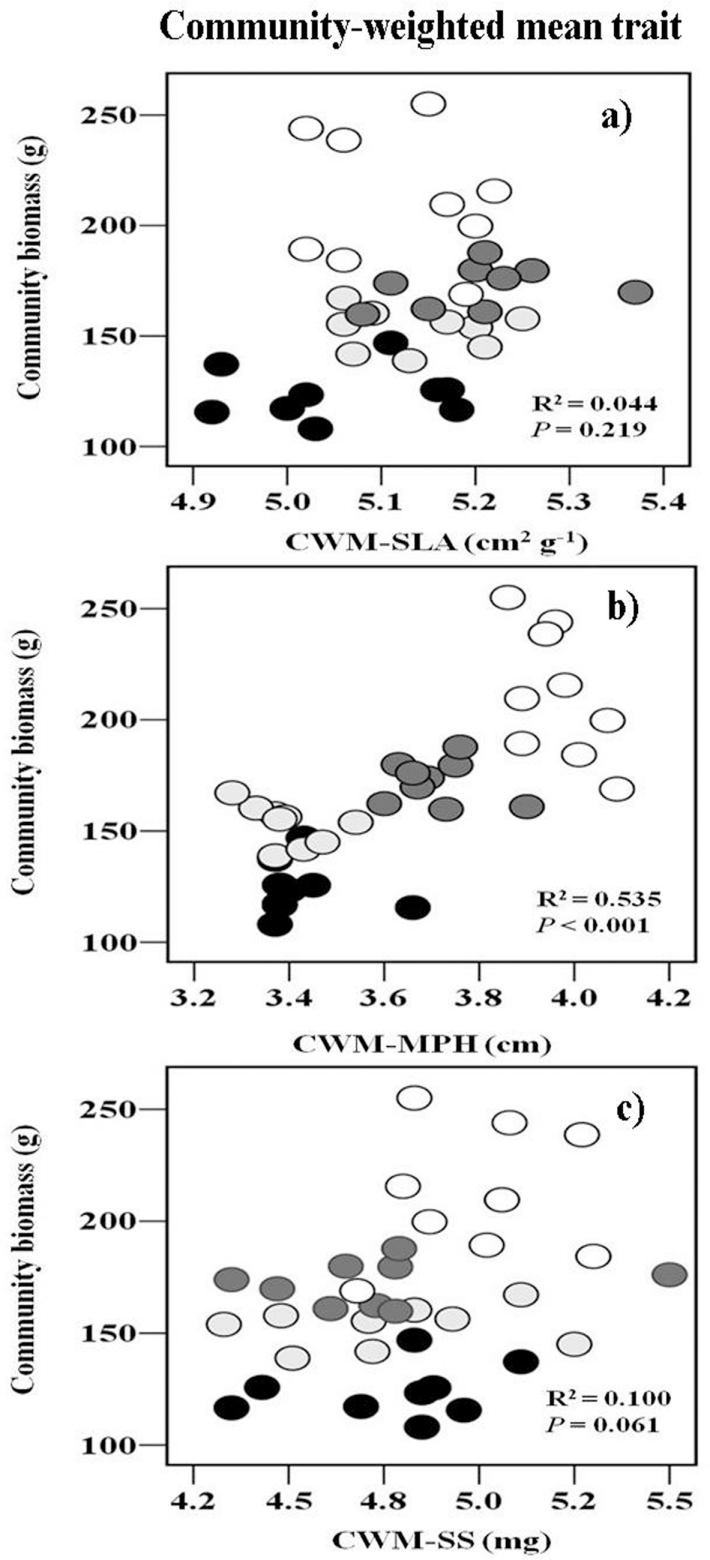
The relationships between community biomass(n = 9) and CWM trait. R^2^and P value was estimated from a linear regression model. CWM, community-weighted mean trait values; SLA, leaf area per unit dry mass; MPH, mature plant height; SS, seed size.

As expected, we found significant negative correlations between community biomass and species richness (Fig 3A), Shannon-Wiener diversity (Fig 3B), and species evenness (Fig 3C). FRic, FEve and FDao did not affect community biomass production (Fig 3D, 3E and 3F). There was a significantly positive correlation between community biomass and CWM-MPH (Fig 4B), but not a significant correlation between community biomass and CWM-SLA (Fig 4A) and CWM-SS (Fig 4C).

## Discussion

The results of our present grassland experiments complement past work on diversity loss due to fertilization by comparing different measurements of plant diversity (species diversity and functional diversity) along a fertilization level, and its relationship with productivity in an alpine meadow community. Our results emphasized that community productivity is linked to the functional traits of the dominant species rather than functional diversity, and also supported the mass ratio hypothesis.

Previous studies have often used species diversity as a proxy for functional diversity in examining the functional consequences of species loss following land use changes. Our results showed that functional diversity and species diversity were independent of each other in this alpine meadow, and they had different responses to short-term fertilization. Similar to other studies, species richness and the Shannon-Wiener diversity index significantly declined along increased fertilization levels [41, 42]. In addition, the grassland had been overgrazed until the beginning of the experiment (4 years before the measurements), and this suggested that the plant community has already been subjected to intense pressure selecting for species that can cope with high grazing intensity and high nutrient loadings from the animal manure, therefore the species evenness was and remained higher. In contrast, the functional diversity (FRic, FEve, and FRao) did not vary similarly to species diversity along the fertilization levels (Fig 1). This showed that the functional differentiation among species’ and species richness can vary rather independently to each other (Table 2), and species diversity may not be good proxy for functional diversity [19].

The changes in functional diversity after fertilization might result from two opposite mechanisms. First, functional diversity may decrease because of competitive exclusion and species loss following fertilization [18, 22]. For example, FRic (volume of the functional space occupied by the community) may be reduced due to habitat filtering [43]. Second, functional diversity may increase because of stronger competition and niche differentiation between persisting species [6, 35]. In this case, competition is likely to limit the similarity of species, and species traits may gradually become divergent in these environments [44]. Ultimately, how functional diversity responds to fertilization depends on a balance between these two antagonistic mechanisms.

In this study, the functional diversity would remain stable because of two opposite processes. On the one hand, this performance of functional diversity may mainly result from the loss of inferior species in fertilized communities, consistent with other studies [32–34]. Species with traits of faster growth rate or greater height will be successful in these environments. As a result, the convex hull volume (the volume of trait space occupied by species in a community) in fertilized plots may be lower when compared to the unfertilized plots [34, 43]. On the other hand, this performance of the functional diversity was closely related with asymmetric changes in functional trait values among species in response to fertilization. The increase in height of dominant grasses was considerably larger than that of rare forbs, as observed in this study. These changes in trait values caused increased niche differentiation between persisting species in fertilized plots. Moreover, this alpine meadow community may have multiple species that play similar functional roles (functional redundancy species). High redundancy can provide insurance and show compensatory responses against the loss of species following fertilization [24, 36, 45]. Our study also showed that there was not a co-variation between species diversity and functional diversity along the fertilization levels, indicating the mechanisms that support the coexistence of many species not necessarily support the functional differentiation among those species [46].

Although functional diversity did not show any change along the fertilization level, CWM traits had a significant increase (SLA and MPH). Our results showed that some grasses species (e.g. *Elymus nutans*, *Poa poophagorum*, and *Koeleria cristata*) would dominate and exclude forbs species in fertilized plots (S1 Table). Although forbs species account for less total biomass than the grass species, they formed the bulk of species richness and diversity in this community, as in most herbaceous communities [20].The decrease of species diversity after fertilization mainly results from the decrease of forbs species. The increased dominance of tall grass species with fertilization suggested that increased competition for light or soil resources resulted in the loss of forbs species. Therefore, changes in the values of CWM traits may result from the replacement of species with traits of low growth rate and low height to species with traits of high growth rate and greater height [47].

In natural communities, previous studies have revealed that there were various patterns of productivity–diversity relationships [3, 48], but fertilization experiments reported nearly ubiquitous negative productivity–diversity relationships [49–51]. Consistent with previous fertilization studies, our results clearly showed that species diversity (species richness and Shannon-Wiener diversity) was negatively correlated with aboveground biomass (Fig 3).

Some studies have shown that an increase in functional diversity leads to more efficiently used light or soil nutrient resources [4, 52], thus facilitating the accumulation of community biomass following fertilization. Inconsistent with these studies, our results demonstrated that there was not a correlation between functional diversity (FRic, FEve and FRao) and community biomass production. This implies that the link between functional diversity and ecosystem functioning might be context dependent, and this dependence might be related to the amplitude of the species’ traits and how species divide into the niche space available [53]. In another way, although functional diversity is insensitive to fertilization in this alpine meadow (high functional redundancy), ecosystem functioning (community biomass) still be maintained at a high level.

As proposed by the “mass ratio hypothesis” [20], the CWM trait values were strongly linked to ecosystem functioning. Our results showed that there was a significant positive correlation between community biomass and CWM-MPH. Our results supported that the functional identities (MPH) of the dominant species largely determine ecosystem functioning and ecosystem functioning is relatively insensitive to the richness of less abundant species [21, 54]. Following fertilization, many forbs species gradually disappeared due to the increased competition for soil and/or light resources, but grasses increased dominance with a higher growth rate and a greater height in fertilized plots [33, 34]. Several other studies have recently found that species richness decreases by increasing the abundance of dominant species or clonal species in response to fertilization [2, 55]. Our finding is consistent with previous studies, which found that the functional identity of dominant species may be the main driver for community biomass production [55, 56].

## Conclusions

Overall, these results suggest that decreases in species diversity due to fertilization do not directly result in corresponding changes in functional diversity, and species composition and functional identity of species may be more important than functional diversity in influencing ecosystem processes (e.g., primary productivity) in this alpine meadow community. Our study also supports the mass ratio hypothesis, that is, the functional traits of the dominant species influenced community productivity. A long-term investigation is further needed to better disentangle the drivers of functional diversity and its consequences for ecosystem functioning.

## Supporting Information

S1 Table Mean ± SE of species relative abundance (SRA) in 9 quadrats and measured specific leaf area (SLA, cm^2^/g, N = 54), mature height (MPH, cm, N = 18) and seed size (SS, mg, N = 3) and in each fertilization gradient. FG, functional group; G, grasses; F, Forbs; L, Legumes; F0, F15, F30 and F60 represent (NH_4_)_2_HPO_4_ fertilizer applications of 0, 15, 30 and 60 g m^-2^ yr^-1^.
(DOCX)
