# Supplementary material for: Plant Functional Diversity Can Be Independent of Species Diversity: Observations Based on the Impact of 4-Yrs of Nitrogen and Phosphorus Additions in an Alpine Meadow
Source: PLoS One. 2015 Aug 21;10(8):e0136040. doi: 10.1371/journal.pone.0136040 (PMC4546594; doi:10.1371/journal.pone.0136040)
Supplement: S1 Table — FG, functional group; G, grasses; F, Forbs; L, Legumes; F0, F15, F30 and F60 represent (NH4)2HPO4 fertilizer applications of 0, 15, 30 and 60 g m-2 yr-1. (DOCX) [file pone.0136040.s001.docx]

**Supporting information**

S1 Table Mean ± SE of species relative abundance (SRA) in 9 quadrats and measured specific leaf area (SLA, cm^2^/g, N=54), mature height (MPH, cm, N=18) and seed size (SS, mg, N=3) and in each fertilization gradient. FG, functional group; G, grasses; F, Forbs; L, Legumes; F0, F15, F30 and F60 represent (NH_4_)_2_HPO_4_ fertilizer applications of 0, 15, 30 and 60 g m^-2^ yr^-1^.

| **Species** |  | **FG** | **SRA** | **SLA** | **MPH** | **SS** |
| --- | --- | --- | --- | --- | --- | --- |
| ***Elymus nutans*** | F0 | G | 0.075±0.015 | 166.008±8.172 | 58.913±2.378 | 240.320±2.381 |
|  | F15 |  | 0.061±0.014 | 182.349±6.791 | 65.093±1.487 | 245.310±1.724 |
|  | F30 |  | 0.090±0.016 | 244.780±17.762 | 74.987±2.127 | 267.210±1.321 |
|  | F60 |  | 0.255±0.020 | 242.998±7.023 | 88.627±3.687 | 300.540±1.231 |
| ***Kobresia capillifolia*** | F0 | G | 0.151±0.030 | 84.825±2.624 | 35.347±1.248 | 143.290±0.598 |
|  | F15 |  | 0.171±0.026 | 84.837±2.079 | 37.087±3.458 | 142.370±0.795 |
|  | F30 |  | 0.127±0.018 | 86.328±2.530 | 46.513±1.289 | 138.630±1.107 |
|  | F60 |  | 0.080±0.016 | 101.441±5.659 | 48.655±3.548 | 140.360±0.967 |
| ***Festuca ovina*** | F0 | G | 0.009±0.003 | 195.342±17.110 | 17.060±1.879 | 28.190±1.278 |
|  | F15 |  | 0.014±0.006 | 190.506±18.976 | 24.067±0.987 | 32.140±0.997 |
|  | F30 |  | **–** | **–** | **–** | **–** |
|  | F60 |  | **–** | **–** | **–** | **–** |
| ***Agrostis hugoniana Rendle*** | F0 | G | 0.019±0.007 | 258.027±13.537 | 38.033±1.247 | 10.450±0.579 |
|  | F15 |  | 0.014±0.005 | 238.759±11.760 | 41.507±2.378 | 10.120±0.796 |
|  | F30 |  | 0.034±0.004 | 269.966±13.678 | 47.960±1.479 | 9.730±0.669 |
|  | F60 |  | 0.037±0.009 | 306.820±12.549 | 49.698±2.367 | 10.280±1.101 |
| ***Poa poophagorum*** | F0 | G | 0.032±0.009 | 177.569±8.904 | 47.080±3.140 | 20.490±2.124 |
|  | F15 |  | 0.062±0.016 | 259.150±11.259 | 46.307±1.279 | 21.350±1.107 |
|  | F30 |  | 0.073±0.015 | 222.318±10.697 | 54.187±2.987 | 19.320±0.968 |
|  | F60 |  | 0.087±0.017 | 247.985±8.621 | 65.450±2.147 | 17.420±1.117 |
| ***Koeleria cristata*** | F0 | G | 0.017±0.004 | 180.861±6.899 | 50.967±1.247 | 16.320±1.017 |
|  | F15 |  | 0.017±0.007 | 192.504±7.328 | 50.073±2.378 | 20.130±0.968 |
|  | F30 |  | 0.040±0.008 | 195.897±8.412 | 49.237±3.147 | 16.170±0.879 |
|  | F60 |  | 0.027±0.010 | 212.896±7.223 | 49.657±1.217 | 15.120±0.974 |
| ***Anemone rivularis*** | F0 | F | 0.046±0.004 | 171.140±3.482 | 39.427±0.998 | 511.420±2.128 |
|  | F15 |  | 0.065±0.025 | 201.268±4.568 | 34.700±2.147 | 538.320±1.976 |
|  | F30 |  | 0.050±0.007 | 204.221±5.784 | 36.107±1.248 | 563.230±1.587 |
|  | F60 |  | 0.076±0.005 | 245.737±6.719 | 44.260±1.369 | 586.640±0.997 |
| ***Sphallerocarpus gracilis*** | F0 | F | 0.022±0.007 | 181.541±4.761 | 22.420±2.178 | 402.820±2.104 |
|  | F15 |  | 0.013±0.005 | 222.640±5.796 | 20.533±0.987 | 423.140±2.017 |
|  | F30 |  | 0.010±0.006 | 247.617±4.128 | 27.013±1.001 | 412.330±1.983 |
|  | F60 |  | 0.068±0.016 | 252.232±6.254 | 27.353±0.897 | 429.240±1.812 |
| ***Anemone obtusiloba*** | F0 | F | 0.041±0.008 | 190.841±3.134 | 22.087±1.278 | 130.210±1.279 |
|  | F15 |  | 0.057±0.007 | 195.484±4.154 | 23.113±0.987 | 135.640±1.107 |
|  | F30 |  | 0.057±0.011 | 210.149±3.547 | 24.373±1.014 | 142.580±0.967 |
|  | F60 |  | 0.055±0.012 | 244.413±4.657 | 24.368±0.657 | 139.650±1.140 |
| ***Anemone trullifolia*** | F0 | F | 0.006±0.003 | 177.137±9.632 | 12.713±0.324 | 251.230±2.001 |
|  | F15 |  | 0.001±0.001 | 199.809±7.741 | 13.933±0.554 | 265.220±1.917 |
|  | F30 |  | **–** | **–** | **–** | **–** |
|  | F60 |  | **–** | **–** | **–** | **–** |
| ***Aster alpinus*** | F0 | F | 0.069±0.015 | 148.630±3.978 | 33.973±1.278 | 31.430±0.987 |
|  | F15 |  | 0.039±0.011 | 170.103±4.472 | 28.773±1.057 | 28.140±0.617 |
|  | F30 |  | **–** | **–** | **–** | **–** |
|  | F60 |  | **–** | **–** | **–** | **–** |
| ***Euphorbia micractina*** | F0 | F | 0.019±0.006 | 309.840±16.407 | 17.320±0.674 | 149.640±1.117 |
|  | F15 |  | 0.044±0.008 | 267.517±14.104 | 14.753±0.873 | 148.320±1.654 |
|  | F30 |  | 0.043±0.008 | 261.575±13.658 | 21.813±1.768 | 142.110±1.017 |
|  | F60 |  | 0.051±0.007 | 335.753±12.487 | 20.695±1.001 | 149.320±1.007 |
| ***Gentianopsis paludosa*** | F0 | F | 0.003±0.001 | 264.227±13.034 | 37.193±2.249 | 12.630±0.987 |
|  | F15 |  | 0.001±0.001 | 314.104±12.413 | 34.413±1.220 | 15.110±0.654 |
|  | F30 |  | **–** | **–** | **–** | **–** |
|  | F60 |  | **–** | **–** | **–** | **–** |
| ***Halenia elliptica*** | F0 | F | 0.019±0.008 | 242.160±11.972 | 27.353±0.987 | 130.410±1.247 |
|  | F15 |  | 0.018±0.007 | 240.170±10.876 | 26.167±0.457 | 132.450±1.017 |
|  | F30 |  | **–** | **–** | **–** | **–** |
|  | F60 |  | **–** | **–** | **–** | **–** |
| ***Delphinium kamaonense*** | F0 | F | 0.014±0.003 | 168.088±14.458 | 35.387±0.667 | 71.450±1.579 |
|  | F15 |  | 0.016±0.004 | 181.651±10.346 | 25.553±1.117 | 52.430±0.789 |
|  | F30 |  | **–** | **–** | **–** | **–** |
|  | F60 |  | **–** | **–** | **–** | **–** |
| ***Saussurea stella*** | F0 | F | 0.033±0.009 | 124.798±15.566 | 15.900±1.214 | 126.430±1.124 |
|  | F15 |  | 0.041±0.016 | 139.584±13.245 | 19.407±1.027 | 123.330±1.078 |
|  | F30 |  | **–** | **–** | **–** | **–** |
|  | F60 |  | **–** | **–** | **–** | **–** |
| ***Thalictrum alpinum*** | F0 | F | 0.009±0.004 | 215.092±15.124 | 14.760±0.367 | 152.350±1.479 |
|  | F15 |  | 0.031±0.010 | 245.806±13.269 | 12.893±0.697 | 142.320±1.981 |
|  | F30 |  | **–** | **–** | **–** | **–** |
|  | F60 |  | **–** | **–** | **–** | **–** |
| ***Ranunculus tanguticus*** | F0 | F | 0.010±0.005 | 231.133±13.496 | 21.780±1.247 | 34.930±1.014 |
|  | F15 |  | 0.026±0.008 | 218.576±10.239 | 21.640±0.478 | 45.120±0.917 |
|  | F30 |  | **–** | **–** | **–** | **–** |
|  | F60 |  | **–** | **–** | **–** | **–** |
| ***Leontopodium nanum*** | F0 | F | 0.016±0.009 | 325.103±19.641 | 16.433±0.998 | 9.890±0.547 |
|  | F15 |  | 0.019±0.012 | 299.973±12.697 | 18.427±0.679 | 10.230±0.499 |
|  | F30 |  | **–** | **–** | **–** | **–** |
|  | F60 |  | **–** | **–** | **–** | **–** |
| ***Trollius farreri*** | F0 | F | 0.010±0.006 | 153.595±14.656 | 22.427±1.247 | 52.430±1.014 |
|  | F15 |  | 0.014±0.007 | 158.567±12.789 | 18.460±1.267 | 30.320±1.210 |
|  | F30 |  | 0.022±0.008 | 170.160±11.324 | 17.447±1.117 | 42.120±0.761 |
|  | F60 |  | **–** | **–** | **–** | **–** |
| ***Potentilla fragarioides*** | F0 | F | 0.029±0.007 | 148.255±15.239 | 23.740±0.937 | 122.620±1.967 |
|  | F15 |  | 0.031±0.006 | 168.612±11.046 | 24.300±1.247 | 132.450±0.967 |
|  | F30 |  | **–** | **–** | **–** | **–** |
|  | F60 |  | **–** | **–** | **–** | **–** |
| ***Plantago depressa*** | F0 | F | 0.006±0.003 | 258.910±10.062 | 6.360±0.217 | 33.280±0.569 |
|  | F15 |  | 0.014±0.012 | 269.912±9.651 | 9.027±0.431 | 29.140±0.754 |
|  | F30 |  | **–** | **–** | **–** | **–** |
|  | F60 |  | **–** | **–** | **–** | **–** |
| ***Saussurea nigrescens*** | F0 | F | 0.068±0.022 | 186.562±5.659 | 25.307±1.117 | 278.230±2.001 |
|  | F15 |  | 0.026±0.012 | 164.143±7.398 | 20.360±1.237 | 227.460±1.874 |
|  | F30 |  | **–** | **–** | **–** | **–** |
|  | F60 |  | **–** | **–** | **–** | **–** |
| ***Geranium pylzowianum*** | F0 | F | 0.003±0.002 | 208.691±6.762 | 11.720±0.964 | 302.330±1.917 |
|  | F15 |  | 0.002±0.001 | 196.163±8.459 | 18.487±1.013 | 330.780±1.547 |
|  | F30 |  | **–** | **–** | **–** | **–** |
|  | F60 |  | **–** | **–** | **–** | **–** |
| ***Allium sikkimense*** | F0 | F | 0.026±0.006 | 143.608±6.378 | 35.426±1.587 | 28.650±0.697 |
|  | F15 |  | 0.033±0.008 | 158.309±8.124 | 37.481±2.001 | 27.890±0.881 |
|  | F30 |  | **–** | **–** | **–** | **–** |
|  | F60 |  | **–** | **–** | **–** | **–** |
| ***Veronoca eriogyne*** | F0 | F | 0.003±0.002 | 227.491±7.896 | 33.789±1.814 | 12.690±0.647 |
|  | F15 |  | 0.012±0.004 | 228.252±10.214 | 35.719±0.927 | 14.570±0.594 |
|  | F30 |  | 0.004±0.003 | 209.813±9.347 | 39.783±1.217 | 12.680±1.001 |
|  | F60 |  | **–** | **–** | **–** | **–** |
| ***Taraxacum maurocarpum*** | F0 | F | 0.013±0.006 | 213.774±8.456 | 34.568±0.367 | 51.230±1.471 |
|  | F15 |  | 0.007±0.003 | 257.163±7.154 | 30.128±2.001 | 52.360±1.347 |
|  | F30 |  | **–** | **–** | **–** | **–** |
|  | F60 |  | **–** | **–** | **–** | **–** |
| ***Oxytropis ochrocephala*** | F0 | L | 0.012±0.004 | 184.110±5.647 | 33.678±2.171 | 149.650±1.687 |
|  | F15 |  | 0.023±0.005 | 222.316±7.169 | 30.214±1.367 | 149.320±1.217 |
|  | F30 |  | **–** | **–** | **–** | **–** |
|  | F60 |  | **–** | **–** | **–** | **–** |
| ***Astragalus polycladus*** | F0 | L | 0.020±0.006 | 242.121±10.387 | 21.368±1.017 | 163.540±1.347 |
|  | F15 |  | 0.013±0.007 | 273.728±6.871 | 20.125±0.997 | 168.360±1.547 |
|  | F30 |  | **–** | **–** | **–** | **–** |
|  | F60 |  | **–** | **–** | **–** | **–** |
| ***Thermopsis lanceolata*** | F0 | L | 0.037±0.013 | 165.909±8.479 | 21.607±0.938 | 1913.220±2.101 |
|  | F15 |  | 0.030±0.011 | 203.569±10.297 | 21.793±1.124 | 1944.590±1.917 |
|  | F30 |  | 0.038±0.012 | 203.813±11.031 | 23.513±0.894 | 1920.230±1.201 |
|  | F60 |  | **–** | **–** | **–** | **–** |

Note: “**–**”represents without this species in the correspondent fertilization gradients.
